# Time series of diabetes attributable mortality from 2008 to 2017

**DOI:** 10.1007/s40618-021-01549-w

**Published:** 2021-09-30

**Authors:** U. Fedeli, E. Schievano, S. Masotto, E. Bonora, G. Zoppini

**Affiliations:** 1 Epidemiological Department, Azienda Zero, Veneto Region, Italy; 2 Endocrinology, Diabetes and Metabolism, Department of Medicine, University and Hospital Trust of Verona, Università di Verona, Piazzale Stefani, 1, 37126 Verona, Italy

**Keywords:** Diabetes, Mortality, Time series, Underlying cause of death

## Abstract

**Purpose:**

Diabetes is a growing health problem. The aim of this study was to capture time trends in mortality associated with diabetes.

**Methods:**

The mortality database of the Veneto region (Italy) includes both the underlying causes of death, and all the diseases mentioned in the death certificate. The annual percent change (APC) in age-standardized rates from 2008 to 2017 was computed by the Joinpoint Regression Program.

**Results:**

Overall 453,972 deaths (56,074 with mention of diabetes) were observed among subjects aged ≥ 40 years. Mortality rates declined for diabetes as the underlying cause of death and from diabetes-related circulatory diseases. The latter declined especially in females − 4.4 (CI 95% − 5.3/− 3.4), while in males the APC was − 2.8 (CI 95% − 4.0/− 1.6).

**Conclusion:**

We observed a significant reduction in mortality during the period 2008–2017 in diabetes either as underlying cause of death or when all mentions of diabetes in the death certificate were considered.

**Supplementary Information:**

The online version contains supplementary material available at 10.1007/s40618-021-01549-w.

## Introduction

Diabetes prevalence is rapidly growing worldwide [1]. This disease is an important cause of death. It has been estimated that diabetes will be the seventh leading cause of death worldwide by 2030 [2]. Nevertheless, routinely collected mortality statistics for diabetes are difficult to interpret because of changing prevalence and under-reporting of diabetes as a contributing cause listed in death certificates [3].

An important aspect of mortality is to study the time trends to better estimate the impact of diabetes on the population health. In particular, data on trends of mortality in diabetes may highlight changes over time in preventive measures, therapy and health systems. The most important result in studies evaluating trends in mortality in diabetes from other countries, such as United States [4], Australia [5] and England [6], is the decline over the past 4 decades in death rates for vascular diseases. These changes are attributed to improvements in treatments and risk factors reduction. No such data are available in Southern Europe.

Therefore, in the present study, diabetes was examined both as underlying and as multiple causes of death (any mention in death certificates, irrespective of its selection as the underlying cause), with the aim of capturing time trends in mortality associated with diabetes.

## Methods

The study was carried out in the Veneto region (northeastern Italy), that has about 4.9 million inhabitants. A copy of death certificates of each resident in the Veneto region is routinely transmitted to the Regional Epidemiology Department for coding of the causes of death according to the International Classification of Diseases, 10th Edition (ICD-10). Since 2008 the regional mortality database includes not only the underlying cause of death (UCOD), but also all the diseases mentioned in the certificate, and the selection of the UCOD is performed by means of the Automated Classification of Medical Entities (ACME) software, a tool developed by the National Center for Health Statistics to standardize assignment of the underlying cause [7]. ICD-10 codes for diabetes (E10-E14) were searched for in any position of the death certificates of subjects aged ≥ 40 years in the whole mortality archive through the period 2008 to 2017 to retrieve all diabetes-related deaths. Diabetes-related deaths were analyzed as a whole, and classified according to the following mutually exclusive categories based on the UCOD selected by the ACME software: diabetes; circulatory diseases (ICD-10 codes I00-I99); other causes of death. Furthermore, all-cause mortality in the Veneto region and mortality from circulatory diseases without the mention of diabetes were analyzed to provide the context of time trends in diabetes-related mortality.

Age-truncated (40 years or more) standardized rates were obtained by the direct standardization method, taking the European standard population as reference. Rates were computed for the above causes by gender and calendar year. Population data by gender, age class and calendar year were downloaded from the Italian National Institute of Statistics website (http://demo.istat.it/). Time trends were investigated by means of the Joinpoint Regression Program [8]; the annual percent change in rates (APC) with 95% confidence interval (CI) was assessed by log-linear models, with calendar year as the predictor variable.

## Results

**Fig. 1:**
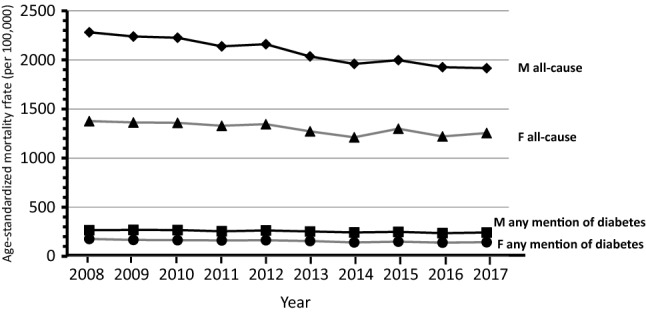
Shows the trends overtime (2008–2017) by gender from all causes and from any mention of diabetes in the death certificate

**Fig. 2:**
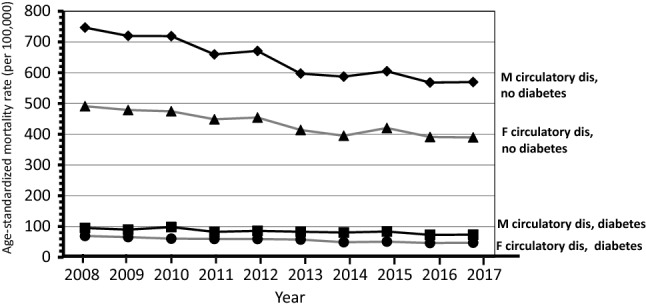
Shows the trends overtime (2008–2017) by gender of mortality from circulatory diseases with and without mention of diabetes

**Fig. 3:**
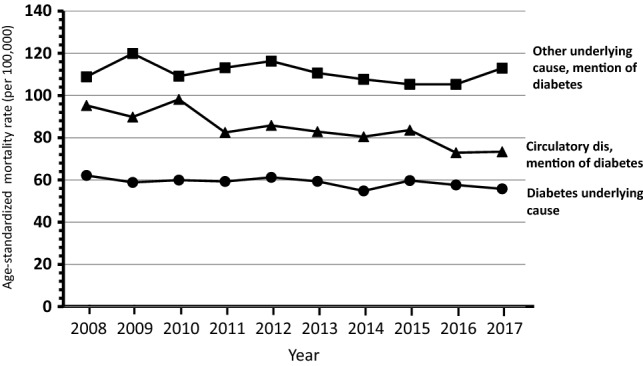
Trends of mortality (2008–2017) from diabetes as the underlying cause of death and from diabetes-related circulatory diseases in males

**Fig. 4:**
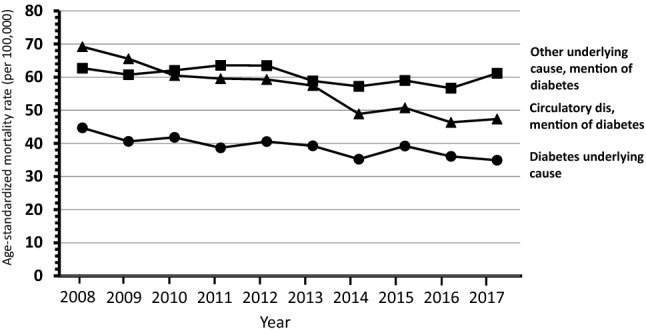
Trends of mortality (2008–2017) from diabetes as the underlying cause of death and from diabetes-related circulatory diseases in females

Overall 453,972 deaths (56,074 with mention of diabetes) were observed among subjects aged ≥ 40 years through the 10-year study period (supplementary table). The trends over time of mortality from all causes and from diabetes considered as any mention in the death certificate, and mortality from circulatory diseases with and without mention of diabetes shown in Figs. 1 and 2 indicate a progressive decline of mortality rates in northeastern Italy. Similar trends were observed for mortality from diabetes as the underlying cause of death and from diabetes-related circulatory diseases that showed a reduction in rates through the study period, while the trend for others underlying causes with the mention of diabetes did not show a clear pattern of reduction in mortality (Figs. 3 and 4).

The APC of mortality from all causes was − 2.1 (CI 95% − 2.5/− 1.7) for males and − 1.3 (CI 95% − 2.0/− 0.6) for females. Mortality from any mention of diabetes showed an APC of − 1.4 (CI 95% − 1.9/− 0.9) for males and − 2.5 (CI 95% − 3.2/− 1.7) for females. The largest decline in mortality was observed for circulatory diseases with diabetes in females − 4.4 (CI 95% − 5.3/− 3.4), while in males was − 2.8 (CI 95% − 4.0/− 1.6); the corresponding figures for subjects without diabetes were − 2.7 (CI 95% − 3.5/− 2.0) for females and − 3.2 (CI 95% − 4.0/− 2.4) for males. The reduction in mortality was significant also for diabetes as underlying cause of death: males − 0.8 (CI 95% − 1.6/− 0.1) and females − 2.2 (CI 95% − 3.3/− 1.2). Death from other causes with mention of diabetes did not show a change over time.

Among diabetes-related deaths, diabetes itself was selected as the UCOD in 24%, and such share remained constant through the study period; by contrast the percentage of deaths assigned to circulatory diseases declined from 37 to 32%. Other diseases were increasingly selected as the UCOD among deaths with mention of diabetes, namely common infections (including pneumonia, from 4 to 7%), and neurologic/psychiatric disorders (mainly dementia/Alzheimer, from 5 to 8%, data not shown).

## Discussion

The main result of the present study is the progressive decrease in mortality over the 2008–2017 period. Interestingly, a significant reduction of mortality was observed either when diabetes was the underlying cause of death or when any mention of diabetes (multiple-cause-of-death approach) was considered. Moreover, the largest reduction in mortality was observed for circulatory diseases. Only in the case of death from other causes with mention of diabetes the decrease in mortality was not significant. Our results are in line with data from other studies in different countries that, in general, show a fall in mortality over the past 3 decades, largely reflecting a decline in mortality due to cardiovascular diseases, cancer and diabetes [4–6, 9–13]. However, not all studies reported a reduction of mortality [14, 15]. Namely, our results are consistent with recent findings from the US obtained by a similar multiple causes of death approach: diabetes-related mortality declined, with a decreasing share of cardiovascular diseases among deaths with the mention of diabetes [16].

Trends over time are informative, as they allow, among other factors, to highlight whether changes in the treatment of both diabetes and complications are effective. Moreover, it is informative also the comparison with the mortality trends in subjects without diabetes: therapy of cardiovascular risk factors may behave differently in subjects with and without diabetes. Moreover, lately new therapy for diabetes, such GLP-1 agonist and SGLT-2 inhibitors, with a recognize beneficial effect on cardiovascular and renal morbidity and mortality are available [17]. These new drugs may further contribute to reduce cardiovascular mortality.

A limitation of our study is that we lacked information on diabetes type, thus we cannot differentiate trends of mortality in type 1 and type 2 diabetes. A further limitation is that data are limited to 2017; however, the whole study period has been coded with the same methodology, assuring the highest level of comparability of mortality records. Moreover, the reduction of circulatory causes and the relative increase in other causes among deaths with diabetes seem to represent a long-term trend already reported from the US and now confirmed also in Italy.

In conclusion, we observed a significant reduction in mortality during the period 2008–2017 in diabetes either as underlying cause of death or when all mentions of diabetes are considered. The largest annual reduction was observed in mortality from cardiovascular diseases in females. By contrast, no significant time trend was observed in mortality rates for deaths with mention of diabetes and a non-circulatory cause selected as the UCOD.

## Supplementary Information

Below is the link to the electronic supplementary material.

Supplementary file1 (DOCX 26 KB)
