# Supplementary material for: Time series of diabetes attributable mortality from 2008 to 2017
Source: J Endocrinol Invest. 2021 Sep 30;45(2):275–8. doi: 10.1007/s40618-021-01549-w (PMC8783866; doi:10.1007/s40618-021-01549-w)
Supplement: Supplementary file 1 — Supplementary file1 (DOCX 26 KB) [file 40618_2021_1549_MOESM1_ESM.docx]

**Supplementary Table**. Number of deaths, crude rates, age-standardized rates by year, gender and cause classified according to diabetes or circulatory diseases (Circ) selected as underlying cause of death (UCOD), or any mention in the death certificate (multiple causes of death, MCOD).

| **number of deaths, ≥40 yrs** | **Males** |  |  |  |  |  | **Females** |  |  |  |  |  |
| --- | --- | --- | --- | --- | --- | --- | --- | --- | --- | --- | --- | --- |
| Year | All causes | Diab UCOD | Diab MCOD | Circ UCOD, Diab MCOD | Circ UCOD, No Diab | Other UCOD, Diab MCOD | All causes | Diab UCOD | Diab MCOD | Circ UCOD, Diab MCOD | Circ UCOD, No Diab | Other UCOD, Diab MCOD |
| 2008 | 20733 | 582 | 2524 | 867 | 6196 | 1075 | 22467 | 743 | 2933 | 1165 | 8255 | 1025 |
| 2009 | 20715 | 555 | 2559 | 839 | 6090 | 1165 | 22509 | 680 | 2828 | 1124 | 8110 | 1024 |
| 2010 | 20791 | 589 | 2581 | 896 | 6171 | 1096 | 22880 | 720 | 2838 | 1065 | 8198 | 1053 |
| 2011 | 20844 | 594 | 2581 | 818 | 5942 | 1169 | 23344 | 694 | 2908 | 1105 | 8184 | 1109 |
| 2012 | 21622 | 630 | 2738 | 882 | 6231 | 1226 | 24376 | 757 | 3013 | 1118 | 8630 | 1138 |
| 2013 | 21240 | 624 | 2702 | 854 | 5842 | 1224 | 23815 | 751 | 2971 | 1124 | 8206 | 1096 |
| 2014 | 21258 | 616 | 2720 | 889 | 5998 | 1215 | 23508 | 704 | 2782 | 996 | 8143 | 1082 |
| 2015 | 22367 | 684 | 2861 | 939 | 6374 | 1238 | 25989 | 793 | 2999 | 1054 | 8972 | 1152 |
| 2016 | 22144 | 674 | 2758 | 833 | 6202 | 1251 | 24847 | 754 | 2874 | 992 | 8511 | 1128 |
| 2017 | 22571 | 665 | 2917 | 867 | 6351 | 1385 | 25952 | 734 | 2997 | 1022 | 8653 | 1241 |
|  |  |  |  |  |  |  |  |  |  |  |  |  |
| **crude rate x100000, ≥40 yrs** | **Males** |  |  |  |  |  | **Females** |  |  |  |  |  |
| Year | All causes | Diab UCOD | Diab MCOD | Circ UCOD, Diab MCOD | Circ UCOD, No Diab | Other UCOD, Diab MCOD | All causes | Diab UCOD | Diab MCOD | Circ UCOD, Diab MCOD | Circ UCOD, No Diab | Other UCOD, Diab MCOD |
| 2008 | 1687,4 | 47,4 | 205,4 | 70,6 | 504,3 | 87,5 | 1638,1 | 53,6 | 211,4 | 83,9 | 594,6 | 73,8 |
| 2009 | 1655,9 | 44,4 | 204,6 | 67,1 | 486,8 | 93,1 | 1613,5 | 48,2 | 200,5 | 79,6 | 574,6 | 72,6 |
| 2010 | 1635,3 | 46,3 | 203,0 | 70,5 | 485,4 | 86,2 | 1616,5 | 50,3 | 198,3 | 74,4 | 572,4 | 73,5 |
| 2011 | 1617,0 | 46,1 | 200,2 | 63,5 | 461,0 | 90,7 | 1623,9 | 47,8 | 200,3 | 76,1 | 563,6 | 76,4 |
| 2012 | 1658,3 | 48,3 | 210,0 | 67,6 | 477,9 | 94,0 | 1677,9 | 51,6 | 205,6 | 76,2 | 588,2 | 77,6 |
| 2013 | 1602,1 | 47,1 | 203,8 | 64,4 | 440,6 | 92,3 | 1617,8 | 50,5 | 200,0 | 75,6 | 552,1 | 73,7 |
| 2014 | 1566,4 | 45,4 | 200,4 | 65,5 | 442,0 | 89,5 | 1564,5 | 46,5 | 183,7 | 65,7 | 537,3 | 71,4 |
| 2015 | 1625,1 | 49,7 | 207,9 | 68,2 | 463,1 | 90,0 | 1708,5 | 51,7 | 195,8 | 68,7 | 585,2 | 75,1 |
| 2016 | 1593,9 | 48,5 | 198,5 | 60,0 | 446,4 | 90,0 | 1621,9 | 48,8 | 186,2 | 64,2 | 551,1 | 73,0 |
| 2017 | 1609,6 | 47,4 | 208,0 | 61,8 | 452,9 | 98,8 | 1680,3 | 47,2 | 192,8 | 65,7 | 556,2 | 79,8 |
|  |  |  |  |  |  |  |  |  |  |  |  |  |
| **age-stand rate x100000, ≥40 yrs** | Males |  |  |  |  |  | Females |  |  |  |  |  |
| Year | All causes | Diab UCOD | Diab MCOD | Circ UCOD, Diab MCOD | Circ UCOD, No Diab | Other UCOD, Diab MCOD | All causes | Diab UCOD | Diab MCOD | Circ UCOD, Diab MCOD | Circ UCOD, No Diab | Other UCOD, Diab MCOD |
| 2008 | 2281,3 | 62,1 | 266,4 | 95,3 | 746,7 | 108,9 | 1376,7 | 44,7 | 176,6 | 69,2 | 491,2 | 62,7 |
| 2009 | 2238,7 | 58,9 | 268,6 | 89,8 | 719,7 | 119,9 | 1363,4 | 40,6 | 166,9 | 65,5 | 478,7 | 60,7 |
| 2010 | 2225,9 | 59,9 | 267,3 | 98,2 | 718,9 | 109,2 | 1359,1 | 41,8 | 164,4 | 60,5 | 474,6 | 62,1 |
| 2011 | 2138,3 | 59,3 | 255,0 | 82,5 | 659,6 | 113,2 | 1329,4 | 38,7 | 161,8 | 59,6 | 448,5 | 63,6 |
| 2012 | 2160,4 | 61,2 | 263,4 | 85,8 | 670,8 | 116,3 | 1345,5 | 40,6 | 163,3 | 59,3 | 454,3 | 63,5 |
| 2013 | 2035,4 | 59,4 | 253,0 | 82,9 | 597,2 | 110,7 | 1272,5 | 39,3 | 155,6 | 57,5 | 413,8 | 58,9 |
| 2014 | 1959,3 | 54,8 | 243,0 | 80,5 | 587,3 | 107,7 | 1211,8 | 35,2 | 141,3 | 48,9 | 395,0 | 57,2 |
| 2015 | 1997,6 | 59,7 | 248,7 | 83,6 | 604,7 | 105,3 | 1299,5 | 39,2 | 149,0 | 50,7 | 420,3 | 59,0 |
| 2016 | 1926,1 | 57,6 | 235,8 | 72,9 | 568,0 | 105,3 | 1220,8 | 36,1 | 139,1 | 46,4 | 391,0 | 56,7 |
| 2017 | 1916,3 | 55,8 | 242,2 | 73,4 | 569,5 | 113,0 | 1255,4 | 34,9 | 143,4 | 47,4 | 389,6 | 61,2 |
